# Identification and validation of sRNAs in *Edwardsiella tarda *S08

**DOI:** 10.1371/journal.pone.0172783

**Published:** 2017-03-07

**Authors:** Yuying Sun, Jiquan Zhang, Lei Qin, Cui Yan, Xiaojun Zhang, Dandan Liu

**Affiliations:** 1 College of Marine Life and Fisheries, Huaihai Institute of Technology, Lianyungang, China; 2 Jiangsu Marine Resources Development Research Institute, Lianyungang, China; 3 Co-Innovation Center of Jiangsu Marine Bio-industry Technology, Huaihai Institute of Technology, Lianyungang, China; 4 Key Laboratory of Experimental Marine Biology, Institute of Oceanology, Chinese Academy of Sciences, Qingdao, China; Universiti Putra Malaysia, MALAYSIA

## Abstract

Bacterial small non-coding RNAs (sRNAs) are known as novel regulators involved in virulence, stress responsibility, and so on. Recently, a lot of new researches have highlighted the critical roles of sRNAs in fine-tune gene regulation in both prokaryotes and eukaryotes. *Edwardsiella tarda* (*E*. *tarda*) is a gram-negative, intracellular pathogen that causes edwardsiellosis in fish. Thus far, no sRNA has been reported in *E*. *tarda*. The present study represents the first attempt to identify sRNAs in *E*. *tarda* S08. Ten sRNAs were validated by RNA sequencing and quantitative PCR (qPCR). ET_sRNA_1 and ET_sRNA_2 were homolous to tmRNA and GcvB, respectively. However, the other candidate sRNAs have not been reported till now. The cellular abundance of 10 validated sRNA was detected by qPCR at different growth phases to monitor their biosynthesis. Nine candidate sRNAs were expressed in the late-stage of exponential growth and stationary stages of growth (36~60 h). And the expression of the nine sRNAs was growth phase-dependent. But ET_sRNA_10 was almost expressed all the time and reached the highest peak at 48 h. Their targets were predicted by TargetRNA2 and each sRNA target contains some genes that directly or indirectly relate to virulence. These results preliminary showed that sRNAs probably play a regulatory role of virulence in *E*. *tarda*.

## Introduction

*Edwardsiella tarda* (*E*. *tarda*) is a common and important pathogen of freshwater and marine fish, which causes enormous economic losses to the world-wide aquaculture industry. The pathogenesis of *E*. *tarda* has been studied for a long time and the virulence factors include type III and type VI secretion systems (T3SS and T6SS) [1, 2], chondroitinase [3], nucleoid-associated protein [4], catalase [5], hemolysins [6, 7], flagella [8, 9], adhesion [10], sigma factors RpoN and RpoS [11] and quorum sensing [12, 13].

But the fundamental pathogenic mechanism of *E*. *tarda* still remains to be discovered. In recent years, some significant experimental and theoretical evidence suggested that small non-coding RNAs (sRNAs) could coordinate virulence gene regulations and pathogen survival during infecting the host [14–17]. At the same time, sRNAs are crucial players of regulatory cascades, coordinating the expression of virulence genes in response to environmental or other changes [16, 17]. They are able to adapt the expression of virulence genes to stress and metabolic requirements [17]. These sRNAs function either directly on virulence genes and/or on regulators of virulence genes [16].

While sRNAs have been well known for some time and some examples have been confirmed in *Escherichia coli* and other pathogenic bacteria [18–22], our knowledge of the networks involving sRNAs and controlling pathogenesis in *E*. *tarda* is still in its infancy. Here, we systematically identify sRNAs in *E*. *tarda* genome by RNA sequencing and bioinformatics prediction for the first time. Then, the cellular abundance of validated sRNA was detected by quantitative PCR (qPCR) at different growth phases to monitor their biosynthesis. In addition, the potential targets of sRNAs were also predicted by bioinformatics analysis. Our results will provide insight into the knowledge of virulence regulation of *E*. *tarda* and pave the way for eradicating edwardsiellosis.

## Materials and methods

### Ethics statement

*E*. *tarda* S08 (Accession no. KX279865) was isolated from diseased turbot. Disease outbreaks occurred on some marine turbot farms in Qingdao, China. The farm owners hoped us to determine the causative agents of these outbreaks and assess potential therapies for the treatment of these infections. So they provided a large number of diseased turbot to us for the study. This experiment as described was carried out in strict accordance with the approval of the Animal Care and Use Committee of the Institute of Oceanology, Chinese Academy of Sciences.

### Bacterial strains and growth conditions

*E*. *tarda* S08 isolated from diseased turbot was used for most experiments. The strain was routinely cultured in Tryptic Soy Broth (TSB, Difco) or TSA medium supplemented with additional 1% NaCl at 28°C, 180 rpm. Colistin was added at a final concentration with 12.5 μg/mL when necessary. The growth in the TSB was determined by spectrophotometric values (OD540 nm) at the interval of 2 h. Then, the growth curve was plotted using optical density against time points (2 h, 4 h, ……, 72 h). While the cultures of series of time points at the interval of 6 h were collected for the next step experiments. All the samples were run in triplicate.

### *In silico* prediction of sRNAs

The genome sequences of *E*. *tarda* S08 (data unpublished) and *E*. *tarda* EBI202 (Accession no. CP002154.1) were chosen for *in silico* prediction. The computational methods were applied for the prediction of sRNAs including sRNAscanner and sRNAPredict3. sRNAPredict3 identified sRNAs based on intergenic conservation and Rho-independent terminators in the closely related bacterial genomes. sRNAscanner computes the locations of the intergenic signals using the Positional Weight Matrix (PWM) strategy for the search of intergenic sRNAs. All the parameters were set as the default analytical criteria for the two methods.

### sRNA extraction and RNA sequencing

*E*. *tarda* S08 was grown in TSB medium at 28°C and harvested with centrifugation (at 6, 000×g for 5 min) at the series of time points. Finally, all the samples from different time points were mixed together at equal volumes. The sRNAs were isolated from cell pellets with bacterial small RNA isolation kit (OMEGA, USA). All RNA was treated with RNase free DNase I and library was built for Illumina Hiseq 2000 platform with library constructions kit following the manufacturer’s protocol.

### Promoter prediction and *in silico* validation of predicted sRNAs

The program BPROM was used to predict the promoters of the bacterial sRNAs. The promoter prediction was conducted to search 200 bp upstream of the sRNA start site. RNAfold program was used to carry out the secondary structure prediction based on the lowest folding energy. The sRNAs were blasted into Rfam database to assess the novelty.

### Quantitative PCR assays

Total RNA was extracted using Trizol reagent (Life tech, USA) and then reverse transcribed using oligo dT and random mix primers (ToYoBo, Japan) according to the manufacturer’s protocol. Quantitative PCR was performed to validate the reliability of predicted sRNAs and check the expression abundance of the validated sRNAs at the different growth phages. The qPCR primer pairs for the 10 candidate sRNAs were designed using Primer Premier 6.0. 16 S ribosomal RNA gene was used as internal control for normalization of gene expression. Quantitative PCR was run on Bio-Rad CFX (USA) with initial denaturation of 3 min at 95°C and a subsequent run of 40 cycles each comprising 10 s at 95°C, 10 s at 62°C, and melt curve was performed to assess the primer specificity. The samples were run in triplicate. The 2^-ΔΔCq^ method (relative quantization) was used in which Cq value (threshold cycle) was normalized to endogenous reference gene 16S (ΔCq = Cqtarget—Cqreference) [23]. Using student’s t test, data were considered statistically significant when *p* < 0.05.

### Target prediction of validated sRNAs

Web-based program TargetRNA2 was used to predict the target genes for each validated sRNA. TargetRNA2 considers each mRNA in the replicon as a possible target of the sRNA. 80 bp before the start codon and 20 bp after the start codon were searched. After searching all mRNAs in the specified replicon for interactions with the sRNA, TargetRNA2 outputs a list of likely regulatory targets ranked by *p*-value.

## Results

### Bacterial growth condition of *E*. *tarda* S08

**Fig 1:**
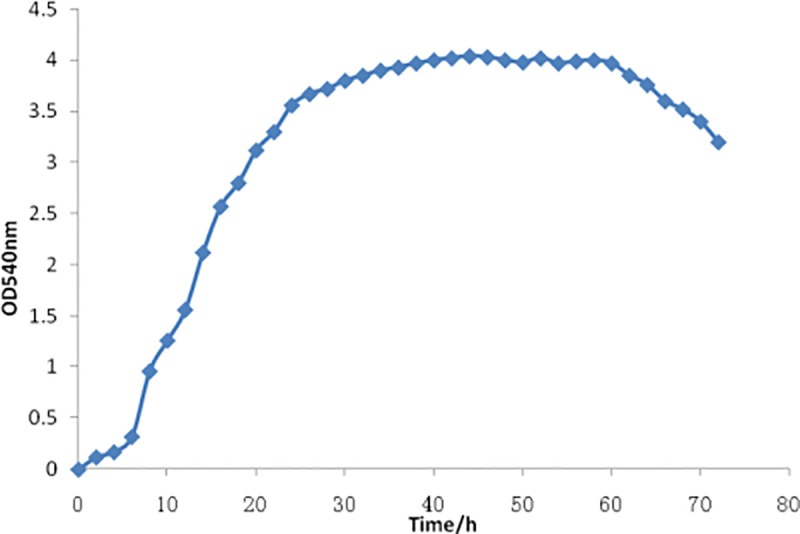
Growth curve of *E*. *tarda* S08.

*E*. *tarda* S08 was cultured in TSB medium at 28°C, 180 rpm. The OD_540nm_ value was monitored at the interval of 2 h and the growth curve was plotted (Fig 1). After 24 h, the strain was showed to grow into post-exponential phage and after 40 h into stationary phage. It entered into decline phase after 60 h.

### Bioinformatic prediction of sRNAs and RNA sequencing

Two computational methods were used to predict the sRNAs and the comparative results were provided as follows (Table 1). After aligned the results, a total of 10 sRNA candidates were predicted (>100 bp in length). Genomic location and the orientations of sRNAs were also analyzed. Table 2 categorized a detailed description of the candidate sRNAs.

**Table 1:** The statistic results of predicted sRNAs.

Method	No. of prediction	Average length	Max length	Min length	CRISPR
sRNAPredict3	111	156 bp	363 bp	66 bp	1
sRNAscanner	134	234 bp	560 bp	34 bp	-
RNA sequencing	2668	83 bp	150 bp	50 bp	-

**Table 2:** The feature description of 10 validated sRNAs.

sRNA name	Start position	End position	SRNA length(bp)	Orientations	Up gene name	Down gene name
ET_sRNA_1	2877375	2877738	364	+	small protein B, tmRNA-binding protein	putative integrase
ET_sRNA_2	797781	797578	204	+	cysteine sulfinate desulfinase	DNA-binding transcriptional activator GcvA
ET_sRNA_3	2731396	2731835	440	-	hypothetical protein	potassium-transporting ATPase subunit A
ET_sRNA_4	3081498	3081919	422	+	lipid A biosynthesis palmitoleoyl acyltransferase	outer membrane lipoprotein
ET_sRNA_5	1285062	1285378	317	+	putative DNA-binding transcriptional regulator	lysine transporter
ET_sRNA_6	1726729	1727058	330	+	phage-related protein	hypothetical protein
ET_sRNA_7	1931296	1931693	398	+	hypothetical protein	hypothetical protein
ET_sRNA_8	3480759	3481235	477	-	4-alpha-glucanotransferase	glucose-1-phosphate adenylyltransferase
ET_sRNA_9	284289	284627	339	-	putative tartrate:succinate antiporter	hypothetical protein
ET_sRNA_10	1443879	1444365	487	+	hypothetical protein	transcriptional activator
ET_sRNA_16s-internal	3710745	3712281	376	-	tRNA-Glu	putative GntR-famly transcriptional regulator

### Promoter and second structure analysis of candidate sRNAs

**Fig 2:**
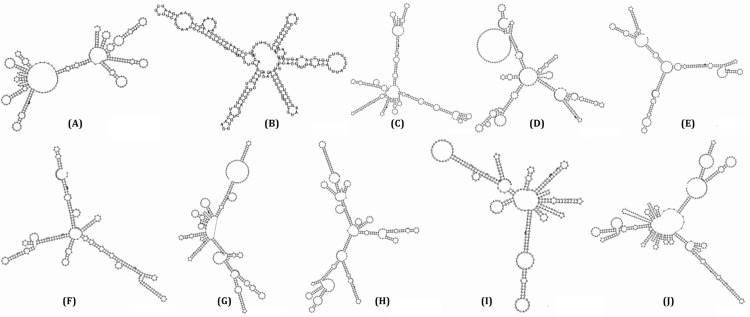
Second structure of ET_sRNA_1~ ET_sRNA_10. **(A)** ET_sRNA_1 (B) ET_sRNA_2 (C) ET_sRNA_3 (D) ET_sRNA_4 (E) ET_sRNA_5 (F) ET_sRNA_6 (G) ET_sRNA_7 (H) ET_sRNA_8 (I) ET_sRNA_9 (J) ET_sRNA_10.

The web-based program, BPRORM, was implemented to perform the promoter analysis. By searching 200 bp upstream of the candidate sRNA start site for the -10 box and -35 box, the results showed that all 10 candidate sRNAs were successfully found the -10 and -35 promoter sites and corresponding TF binding sites. The average distance for the -10 box and -35 box were 53 and 76 bp upstream of the candidate sRNAs, respectively. Secondary structure analysis were carried out using RNAfold program and depicted in Fig 2. Next, the 10 candidate sRNAs were undergone to blast against Rfam database for the novelty. Two of 10 candidate sRNAs, named ET_sRNA_1 and ET_sRNA_2 (homologues to tmRNA and GcvB), showed the homology in Rfam. While the other candidate sRNAs were first found. The sequence of 10 sRNAs genes was analyzed for terminator prediction. Rho-independent terminators were predicted at the 3' end using ARNold (S1 File).

### Experimental validation by qPCR assays under different growth phage

**Fig 3:**
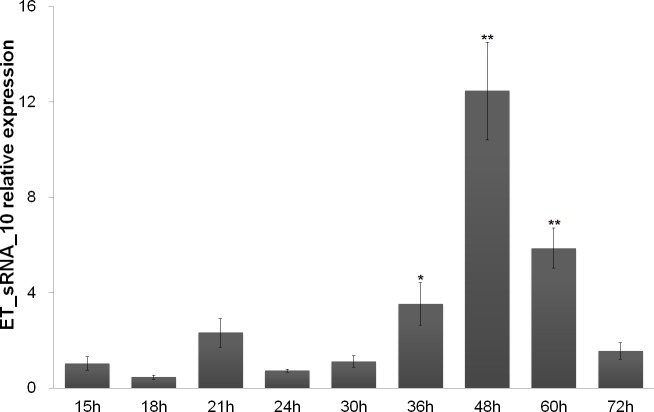
Quantitative PCR detection the transcript levels of ET_sRNA_10 under different growth phases. Statistical significance (*p<0.05;**p<0.01) was obtained using ANOVA test.

Further experimental validation was performed for the 10 candidate sRNAs. The qPCR primer sequences used for sRNA genes were listed in Table 3. The total RNA was extracted from different time points and reverse transcribed. The cDNAs were used as the templates for qPCR to assess the expression of candidate sRNAs. ET_sRNA_10 was almost expressed all the time and reached the highest peak at 48 h (Fig 3). However, the other nine sRNAs were expressed in the late-stage of exponential growth and stationary stages of growth (36~60 h). And their transcript level reached the highest point at the final phase of stationary growth (60 h) (S1–S9 Figs). This showed that the expression of the nine sRNAs was growth phase-dependent.

**Table 3:** The qPCR primer sequences used for sRNA genes.

sRNA name	Primers used for qPCR
ET_sRNA_1	for: 5’actacgcactcgcagcttaataac
	rev: 5’cggacagacacgccactaaca
ET_sRNA_2	for: 5’agacatggcggtggcgtaag
	rev: 5’actaaatcactatggacagacagggta
ET_sRNA_3	for: 5’gcgatagaggacagcaacgataatg
	rev: 5’aaccaacaggagtagcaccagtac
ET_sRNA_4	for: 5’ttacagcatgaagcatcggtcatagaa
	rev: 5’gacggtgagtgagaggaagaggaa
ET_sRNA_5	for: 5’actcgctaataatccgccaaccatc
	rev: 5’tttgtctgagccattagaaccctatcg
ET_sRNA_6	for: 5’cgacctcaagccgaacctcttc
	rev: 5’atgttgccgctgccactacg
ET_sRNA_7	for: 5’accgctggagattccgctatgt
	rev: 5’tgctacaactcactgccgtcac
ET_sRNA_8	for: 5’cgctacccgtttattccagcatcc
	rev: 5’cgcctgtcatccgcaacaaca
ET_sRNA_9	for: 5’catcaggatggtggttctgagtca
	rev: 5’cgccctctttaagtattcccattcaac
ET_sRNA_10	for: 5’cgctgatggatattccgccgatg
	rev: 5’tggtgcttccctctgaacgatagtaa

### Target prediction of validated sRNAs

Accurate prediction of sRNA targets plays an important role in studying sRNA function. The targets of 10 sRNAs were predicted by TargetRNA2 (S2 File). TargetRNA2 outputs a list of likely regulatory targets ranked by *p*-value (*p*≤0.05). A total of 385 potential targets were identified. We parsed the predicted mRNA targets based on their respective protein function (Table 4) [24]. Our result demonstrated that the majority of known targets for sRNAs were involved in metabolism (114), virulence (59), and transport (35). However, a large number of target genes were categorized as ‘other’ (49) and ‘hypothetical proteins’ (115), respectively (Table 4). Each sRNA targets contain a number of genes that directly or indirectly relate to virulence. The result preliminary shows that sRNAs probably play regulatory roles of virulence. Of course, the related work is being verified by experiments.

**Table 4:** sRNA target categorization.

Target classification	Number of predicted targets by category
Cell division	3
Cell wall	5
Metabolism	114
Ribosomal protein	3
Virulence	59
Other	49
Transport	35
Hypothetical protein	115
T3SS	1
T6SS	1
Total	385

Target genes are classified into ten categories based on either known or hypothetical function for *E*. *tarda*.

## Discussion

*E*. *tarda* is associated with edwardsiellosis in cultured fish, resulting in heavy losses in aquaculture. The pathogenesis of *E*. *tarda* has been studied for a long time and some virulence factors have been identified. However, the fundamental pathogenic mechanism of *E*. *tarda* still remains to be discovered. More and more evidence shows that the use of sRNAs is among the strategies developed by bacteria to fine-tune gene expression. They are involved in many biological processes to regulate iron homeostasis [25–27], expression of outer membrane proteins [28, 29], quorum sensing [30, 31], and bacterial virulence [16, 17] through binding to their target mRNAs or proteins.

In this research, it is the first time to report the existence of small RNAs within the genome of *E*. *tarda*. In principle, four major computational methods were applied for the prediction of sRNA locations from bacterial genome sequences: (1) secondary structure and thermodynamic stability, (2) comparative genomics, (3) ‘Orphan’ transcriptional signals and (4) *ab initio* methods regardless of sequence or structure similarity [32]. Transcriptional signal-based sRNA prediction tools include sRNApredict [33], sRNAscanner [34], and sRNAfinder [35]. sRNAPredict depends on the promoter signals, transcription factor binding sites, rho-independent terminator signals predicted by TRANSTERMHP [36] and BLAST [37] outputs as predictive features of sRNAs. sRNApredict3 is recent version of the sRNApredict suite that is used in the efficient prediction of sRNAs, with a high level of specificity. Some researchers found that sRNAPredict provided the best performance by comprehensively considering multiple factors [38]. The main advantage with sRNAscanner is that it uses its own algorithm and the training PWM dataset to calculate the genomic locations of the promoter, transcription factor, and terminator signals. Moreover, the sensitivity and specificity profile of sRNAscanner was first evaluated through the Receiver Operator Characteristic (ROC) curves and confirmed its satisfactory performance [32]. In this research, we choose transcriptional signal-based sRNA prediction tools (sRNA predict3 and sRNA scanner) for *in silico* prediction.

Most of these tools are applied to locate the putative genomic sRNA locations followed by experimental validation of those transcripts. Then 10 sRNAs were validated by RNA sequencing and qPCR, of which 8 novel sRNAs were found. The other two sRNAs, ET_sRNA_1 and ET_sRNA_2, were homolous to tmRNA and GcvB, respectively. TmRNA (also known as 10Sa RNA or SsrA RNA) is a unique bi-functional RNA that acts as both a tRNA and an mRNA to enter stalled ribosomes and direct the addition of a peptide tag to the C terminus of nascent polypeptides. TmRNA is widely distributed among eubacteria and has also been found in some chloroplasts [39]. The sRNA GcvB was first described in *E*. *coli* as being transcribed from a promoter that is divergent from that encoding *gcvA*, which is a transcriptional regulator of the glycine-cleavage-system operon [40–43].

What's more, the cellular abundance of 10 validated sRNA was detected by qPCR at different growth phases to monitor their biosynthesis. ET_sRNA_10 was almost expressed all the time and reached the highest peak at 48 h, which indicated that ET_sRNA_10 was probably house-keeping sRNA. But the expression of the other nine sRNAs was growth phase-dependent and they were expressed in the late-stage of exponential growth and stationary stages of growth. It had been reported that the expression of some sRNAs in gram positive and negative pathogens was growth phase-dependent. The expression of 11 candidate sRNAs was characterized in *Staphylococcus aureus* strains under different experimental conditions, many of which accumulated in the late-exponential phase of growth [44]. The characteristics of 11 sRNAs were studied in *Enterococcus faecalis* V583, six of which were specifically expressed at exponential phase, two of which were observed at stationary phase, and three of which were detected during both phases [45]. The expression of twenty-four sRNAs was also phase- and media- dependent in *Streptococcus pyogenes* M49 [46]. In *Clostridium difficile*, the expression of six sRNAs was growth phase-dependent, three of which (RCd4, RCd5 and SQ1002) were induced at the onset of stationary phase, whereas three of which (RCd2, RCd6 and SQ1498) was high during exponential phase and decreased at the onset of stationary phase [47]. Among the twelve non-coding RNAs found in *Listeria monocytogenes*, two of these non-coding RNAs were expressed in a growth-dependent manner [48]. In *Brucella melitensis*, three validated sRNAs were significantly induced in the stationary phase [49]. In this research, nine sRNAs show growth phase-dependent expression profile. In addition, it has been reported that the expression of some virulence determinants and associated factors in *E*. *tarda* is also growth phase-dependent [50–52]. So, we speculate that some of growth phase-regulated *E*. *tarda* sRNAs may be involved in the control, as previously observed in some gram-positive and gram-negative bacteria [53–55].

Despite the abundance of sRNAs in all bacterial lineages, little is known about their function and mechanism of action within the bacterial genomes and only a few sRNAs have been assigned with functions till date [56]. Using TargetRNA2, we have predicted the target mRNAs of 10 sRNAs.

Functional categorization of the target genes regulated by sRNAs resulted in identification of genes involved in key pathways of cell division, cell wall, transport, virulence,type III secretion system, type VI secretion system, ribosomal protein, and metabolism. A majority of these pathways are critical for the growth and survival of *E*. *tarda* in the host cytoplasm. A significant number (29.87%) of predicted target genes were categorized as ‘hypothetical protein’, which is not surprising considering that nearly 30.89% of *E*. *tarda* EIB202 genes are still reported as hypothetical proteins.

Of course, the related work is being verified by experiments. The mutant strains *E*. *tarda* S08*⊿SsrA*, *E*. *tarda* S08*⊿Gcv* and *E*. *tarda* S08*⊿ET_sRNA_10* have been constructed. The next step is going to verify *in vivo* regulation functions of sRNAs. Once the regulation function of virulence is further confirmed, the unique nature of sRNAs that can be exploited for the development of novel diagnostic tools and therapeutic interventions will maybe come true in the future [57].

## Conclusion

This report presents the study of small non-coding RNAs on *E*. *tarda* for the first time. Ten sRNAs were validated by RNA sequencing and qPCR. ET_sRNA_1 and ET_sRNA_2 were homolous to tmRNA and GcvB, respectively. However, the other candidate sRNAs have not been reported till now. ET_sRNA_10 was almost expressed all the time and reached the highest peak at 48 h. However, the other nine sRNAs were expressed in the late-stage of exponential growth and stationary stages of growth (36~60 h), which showed that their expression was growth phase-dependent. And they probably played regulatory roles during the biological process. The targets of 10 sRNAs were also predicted by TargetRNA2. Each sRNA targets contain some genes that directly or indirectly relate to virulence. These results preliminary showed that sRNAs probably play a regulatory role of virulence in *E*. *tarda*. The related work is being verified by experiments.

## Supporting information

S1 File Sequence analysis of novel sRNAs. The region in yellow and green shows start (5’) and stop (3’) codons respectively. 5’ and 3’ start and ending sites respectively are as predicted by SIPHT/ sRNAPredict3. The region in red shows Rho-independent terminators. The qPCR primer sites are shown in blue.
(PDF)

S2 File Predicted results of 10 sRNAs’ Targets from TargetRNA2. (GZ)

S1 Fig Quantitative PCR detection the transcript levels of ET_sRNA_1 under different growth phases. Statistical significance (*P≤0.05;**P≤0.01) was obtained using Anova test.
(TIF)

S2 Fig Quantitative PCR detection the transcript levels of ET_sRNA_2 under different growth phases. Statistical significance (*P≤0.05;**P≤0.01) was obtained using Anova test.
(TIF)

S3 Fig Quantitative PCR detection the transcript levels of ET_sRNA_3 under different growth phases. Statistical significance (*P≤0.05;**P≤0.01) was obtained using Anova test.
(TIF)

S4 Fig Quantitative PCR detection the transcript levels of ET_sRNA_4 under different growth phases. Statistical significance (*P≤0.05;**P≤0.01) was obtained using Anova test.
(TIF)

S5 Fig Quantitative PCR detection the transcript levels of ET_sRNA_5 under different growth phases. Statistical significance (*P≤0.05;**P≤0.01) was obtained using Anova test.
(TIF)

S6 Fig Quantitative PCR detection the transcript levels of ET_sRNA_6 under different growth phases. Statistical significance (*P≤0.05;**P≤0.01) was obtained using Anova test.
(TIF)

S7 Fig Quantitative PCR detection the transcript levels of ET_sRNA_7 under different growth phases. Statistical significance (*P≤0.05;**P≤0.01) was obtained using Anova test.
(TIF)

S8 Fig Quantitative PCR detection the transcript levels of ET_sRNA_8 under different growth phases. Statistical significance (*P≤0.05;**P≤0.01) was obtained using Anova test.
(TIF)

S9 Fig Quantitative PCR detection the transcript levels of ET_sRNA_9 under different growth phases. Statistical significance (*P≤0.05;**P≤0.01) was obtained using Anova test.
(TIF)
