# Supplementary material for: Identification and validation of sRNAs in Edwardsiella tarda S08
Source: PLoS One. 2017 Mar 7;12(3):e0172783. doi: 10.1371/journal.pone.0172783 (PMC5340389; doi:10.1371/journal.pone.0172783)
Supplement: S1 File — The region in yellow and green shows start (5’) and stop (3’) codons respectively. 5’ and 3’ start and ending sites respectively are as predicted by SIPHT/ sRNAPredict3. The region in red shows Rho-independent terminators. The qPCR primer sites are shown in blue. (PDF) [file pone.0172783.s001.pdf]

> ET_sRNA_1

AGCCACGTGGTGTGCGATCCGACGCGCACCCGTAAGCTGCTGCTCAAGGA

GCGCGAGCTGGCGACCCTGTTCGGCAGCGCCAACCGCGACGGTTACACCA

TCGTCCCGCTGTCGCTGTACTGGAAGAACGCCTGGGCTAAGCTGAAGATC

GGCGTGGCTAAGGGCAAGAAAGAACACGATAAACGCGATGATATCAAGG

CGCGTGAATGGCAGCAGGATAAAGCGCGAATTATGAAAAACGCCGGCCG

CTAACGCTAGCGCAGCGGCGGTAAACTCTGGTATACTGCCGATTACACAC

TTGGGCCTGATTCTGGATTCGACGGGATTTGCGAAACTCAAGGTGCATGC

CGAGGGGCGGTTTGCCTCGTAAAAAGCCGCAAAAAAATAGTCGCAAACG

ACGAAAACTACGCACTCGCAGCTTAATAACCTGCGCAGAGCCCTCTCTCC

CTAGCCTCCGCTCTTAGGACGGGGATCAAGAGAGGTCAAACCCAAAAGAG

ATCGCGTGGAGCTCCTGCCTGGGGGTGAAGCGTTAAAACTAATCAGGCTA

GTTTGTTAGTGGCGTGTCTGTCCGCAGCTGGCAAGCGAATGTAAAGACCA

GACTAAGCATGTAGTACCGACAGCGTAGTAATTTCGGACGCGGGTTCAAC

TCCCGCCAGGTCCACCAAATCATGATCCGGATACGTCCGGTGAAGTACAG

AAAGCCCGCATGGCACAAGCCCTGCGGGCTTTTTTGTGTCTGTCATTGTCC

GAGAACATCCGGCTAAATCCGGTGATTATTGGTATACGTTTAGGTATACG

GTAGGATGTATACCTAAACGCGTATACCAATTCATGAAGGAGCGGCCACA

GTGGCACGGACAACACGCCCCCTTACCAACACCGAAGTTCTGCGTGCTAA

AGCGTTAGAGAAGGATCTAACGCTGCATGATGGCGATGGCCTTTTCCTGA

TAGTGAAAACCAGCGG

> ET_sRNA_2

AGCGGTTGCGCCGGCGGAATAGTTTCAGCCCGAGGAAATCCTCCAAGGAT

TTGATCTGGTGGCTGACCGCGGCCTGCGTCACAAACAGCTCCTCGGCCGC

CTTGGTAAAACTGAGGTGGCGTGCGGCGGCATCAAAGACGCGCAACGCAT

TCAGAGGCGGTAATCGTTTTGACATATGGGATCGCTTATTATCAAATAGTT

ACTATTTACCAACCAATTTGCAACATATTAGTTTTTTTTATCCGAGCCATTA

TAATTTGTCCGTTGAGGATAGACCAGCAAATACCTATAGTGGCGGCAACT

TCCCAAGCCGGAACGAAAAAGCAGGAATGAATCGTTTCAGGAATGCTTTT

GGCTTTGTGGTTGTGATGTTGTGTTTGCAAGTTGTCTGACGCGTTATCAGA

CATGGCGGTGGCGTAAGCCCTGCCGTTTTTCACTTCCTGTACATTTACCCT

GTCTGTCCATAGTGATTTAGTAGCACCGCCTATTGCGGTGCTTTTTTTTTCC

GCTATCGCTGGGACAAAAGCCCACAGAATCGCTTCATCATCGCCGCCCCT

CGGTTTAAAATGCCGGCCATTGAGGCCCCGCGCGCTGCGCCACGCCGCAG

AAGTGGCACCAAATGTGAGCCTGCTCTCGGATTAATCCTGTTAATCCTGGC

AAGGCGATCCACCGCCCTTTCCGCGCCGAATTACTGAGATCCTGCCCATG

ACCTTTGACTTTATGGCCTTGCGCCGCGACTTCCCTGCCCTGGAAACAGAG

GGGGTCTATCTCGACAGCGCGGCCACCGCGCTGAAGCCGCGCGTG

>ET_sRNA_3_rev

TCCCAGCGCAGGCCGGGGAATCCCTGGGGGTTGAGGGGCAGTAGCCCCTG

CGCCATCAGCAGCGCGAACAGCAGCACGATCCCCAACAGGTTAAAGGCG

AGGATGGCCAGCAGATAGTGACGCCAGTCCATTTCATGGGGCGTCACGCC

GCACAGGCGCCACAGGGCGTTCTCCGTACGCGCCATGGCGCGGTACGGCT

CGCCGTCGATCAGGCGGCTCAGCAGGCTGCCCAGCGGTGGGGCCAGAATC

AGCAGCGGCAGCATCAGCAGACCGATTAGCATCAAGGCATGGGTAAACA

TCAGAAGTCCTCCGCATGCCACAGTGCATACACCAGATAAACCAACAGGA

GTAGCACCAGTACGGCACAGGCGATCAGTCCCAGCGTCATTTTTATCTCC

TTTAGCGCGATGACAGTATCAAGATAGGCGCTCTCGGGTATAAATGAGGC

AAAAAGCGGCGTCATCGCTGTAAAAAAAGTATAAAAATGGCGGGGCTGG

CGTGGGGCGCGCCGTGCGTTAGATGCGCGGCGGGAAAGGGGAGGATAGC

CAGGAGACTCTCGCCCATGTCGTGAACGCTCGTCACGCCTGACGATGCGC

CAGCGCGATCGGCATTATCGTTGCTGTCCTCTATCGCATTGCGGCGTACGC

TGACGCAACAAGTGGTCGGAGGTGTGCTTGATCTTTGTGCGCTCAGGGGC

TATGTTGGGGCTGTTTCGGCCACCGTTTTCGGCCGTTTTTTGACGCTCCCG

GCGCCGGCGCCAGCGGACGTATTTGCAAGGAGGTCACGATGTATCAGCGC

TATTCCTGGCCGCGTATCTGGCTGCGCCGCAGCGGCGTGATTCTGGTGGGT

ACCCTGGCCTTTCCCGTGATGCTGCTGCGTCACGATCGGGCACGCTTCTAT

AGCTATCTGCACCGGGTCTGGTCGAAAACCAGCGACAAGCCGGTGTGGCT

GGCGGAGTCAGAAAAGGTTCGGCCCGACTTCTTCTGAGCCCGTGCGCGTC

ACGATACCTGGCAAACCCCATTCGGCAATCTACACTTG

>ET_sRNA_4

TAAGACCCGCCCAGAGGGTATGCCGTCGCTGTATACCCGTTAGGTCACAC

CGCTCACGACATCGTTTACCCGGCAGTCGATATCCTGATATACATCCGCTG

CCGGGCCAACAAACGAAGGCTCAGAGCGACGCTCGGTAATGACGCTTAAT

ACGCTAATTTATACAACATAAAATAACAACACGGAATATAATAGAAGAAA

CCACACAACAGCCATGTAATAAACTGAGAAAACCAAGCCAACATTAAACT

AATTTTTATACTATTGCACCCTCCAGCTTGACCATCATCAAATAACCTAAC

AGTTTATCTATATTGCTAAATTCACGACAAACACATTAACTCTTTTTATAT

ATTACACCTTACCTACCTCAGCGCGGCGAATTAACGCAAATCAATAGTCA

GCAATAGCGCACTAAATCAAATGTTAAGTGTTTTCCAAAGCGCAATGCAC

CAATCTCAGGGTTTACAGCATGAAGCATCGGTCATAGAATCACTTCCGGA

AGATAAAAAGCGTTAAATCCAATCAGTTATAAATTAATCTATCGTTCCTTG

CGGGCACCAGGGACGATAATCAACGCCTAGAATGTAAATGCATCAGCTTT

TATAATCAAACTGGCAGAATAATTCGATTGTCCAATTCCTCTTCCTCTCAC

TCACCGTCGCAGACGGCGAGAGAACTCTTGCGTAACGATATTCCGCCGCG

GCGTCTTGCGCCGTCATGCGTTTTTTGACAGAGAATGGTATATTAAATGAA

ATTTAACACTTCCTGTGCACTGCTGGCAGTCACCTCCTCTTTATTCCTTTCC

GGCTGTATGAATAATGCAGACCAATACGCCGCCGATGTCTATAATCCGAC

GCAGCTAAATCAGAAGCAGGAGAGTAAAACCGTCAATATTATTTCTATTC

TGCCTGCTAAGGTTGCTGTAGATAATAAAGAGAACAAAGAGATGGCTCAG

ACCGTCGGCGCGATCCTGGGCACCGTTGCCGGCGCCGCCGGCGGCTACAA

CCTGGGGCATGGTT

>ET_sRNA_5

GTGAGACCGGCTCCGGCACCCGCGAGGTGGTTGAACATCTGCTGCTTTCC

CATTTACCCCACTTCGATCTGGTGATGGAGCTGGGAAACTCCGAGGCCAT

CAAGCATGCGGTGCGCTATGGCATTGGCATCAGCTGCCTGTCGCGTCGGG

TGATCGAGGAGCAACTGAGCAACGGGACGCTGGTCGAGGTCGCGCTGCCG

GAGATCAACCTGCATCGCTCACTGTATCTGATCCACCACCGCCAGAAGCA

TATCTCCAAGGCGCTGGCCCGTTTTCTGAGCTACTGTCAGGCGTAGCCTCT

GCGACGGCGGCAGCAGAGGCTGCTGCCGTAGCTTATTGTGATCTTTGTGT

ATTTTTATTGCACACTCGATTAGCCACTCGCTAATAATCCGCCAACCATCA

TGAAGCTCTCTTATTATCGTTAGATTGTTACTTCCCGCCGCAGATGGATTT

GTTACAATCCCGCCTGGTTTCATGATTCAACGCAAAACAACGATAGGGTT

CTAATGGCTCAGACAAAAACTACCGTCCCCGACGCGGGACAAGGACCGGC

GCTGCGCCGGGAGTTAAAAGCGCGGCACTTGTCGATGATCGCTATCGGCG

GTTCTATTGGTACTGGGCTGTTTGTCGCATCGGGCGCTACGGTATCCCAGG

CCGGCCCCGGCGGTGCGCTGTTGTCGTATATCATCATCGGGATGATGGTGT

ACTTCCTGATGACCAGCCTGGGTGAGCTGGCGGCGTTTATGCCGGTGTCA

GGTTCGTTTTCTACCTACGGCTCACGCTACGTGGAAGAGGGCTTCGGCTTC

GCGCTGGGCTGGAACTATTGGTATAACTGGGCGGTTACCATCGCCGTCGA

TCTGGTGGCCGCACAGCTGGTGATGGGCTACTGGTTTCCCGATATTCCCGG

CTGGATCTGG

>ET_sRNA_6

ACGTTCGGCGACCTGCGCCTGGGTCAGCCCCGCGTTTTTGCGGGCGGCGA

GCATTTCATCGAGTAGGGCGTATTCCTCTTCGATGGCATCGTATTCTGCTT

TAAATGCCGGGTCTTCCATCCATTTTGCAGCCATTTCATCATGCGTCATCG

TGGGGGGGGTGCGTTTACCAGCCATGTTTTACCTCCTTCATTCTGGTTTCA

GCCTTCTTACGTTCAGCGCTTGGCGTCTTCTGGGTTT

TCTTGATGAAGCTGTGCAGCATGACGATACGTTTTCCGGTAAGAGTGCAG

TAAAACACGCGGGCGATGCCATCACTGCCTTTGATTCGAAGCTCAAAAAG

CCCATCGCCAAAAGGGCTTGTGTGTGGTTCTCCGAGATTACTACCGTAGAT

TTCCATGCGTTCAACGAGATGCTGGTATCGAACCCGCATACCCATTGGCA

ACTGATCGACCTCAAGCCGAACCTCTTCGCTGTAGTACTCAATAGTGTAGT

TCATGGAGTGAAACATAACAATATTGTTATGTTTTCGCAAGTTCTTGCCCA

TCGGAATTGTGAGCGCTAGAGCGTAGTGGCAGCGGCAACATCTGTTGCTG

GGATTGGCGTCCTGGAATGGGCGGTTTTTAAGAGCATTAGGAAATATATG

CCACACACTGTTCCAAGGGGATGGTTATTGCATTCCTATGATTTGTAAGTA

AAGTATCATAAATCGGAATTAGTTTTACCCGCGATGTCGGGGGTATAGTC

CGCTCAGGTGCTGAAAACACCTCAACGCGGCCTCCGCACCCGATAGACTT

GCGGTTTTTTTGTGGTCATTTTTTGGATATGGCCGAGTGTGGGCGAATACA

ATACCAGCGATGGGAATAAGCCCGCTTGACGTTGACAAGTTTTCAGCACT

CGGCCACCCGAGCCGTTCGGGTACCGGGAAGATCG

>ET_sRNA_7

TGGCGCGACGCCTGTAAGATCTGCGTACTGCAGGCGCTGATGCGATCGCG

CTCAGGCAGGGCGTCGCTGTGGGCCAGCCATTGGCCGGCGACGAAGTTTC

GCGCCTGGGCATGCTGAGAGAGATGGCGCATATTGACCACCAGATCCGCC

GCCGTCTGTAACGGGGGAAACGCCTGCGCCTGCGGCACATCGCACTGATA

GCTGCTGCGCAGCGTCTGCGGCACGCTCTCCGTCGCCTGCGGGCCCGCGCT

GCAACCGGCGAGCAGACACACCGCCAGCAGTGAAAGATATTGTTTCATCA

CATATTCCTCGCTAGGTAGGCCGCGATAATAGCAAGCGTACGCCACAACT

CAATGTCTACGGTAAAATTTCCCGACGCAATACAGGACGCCCCCGTTGCG

CAAGGCGATCACGTCCGTGCGCAAAAAAAGAGACAGATCGGTGAGCTGT

ATCACACTCGACCGCTGGAGATTCCGCTATGTTTATTTCATCCGAGGCAAC

GAAAGGAGTTAAATGGCACATTTAATTACGTTCGTGACGGCAGTGAGTTG

TAGCACCCAACGGACCCTCGTCTTGGCCAGCCCGGAATATGCCACTGAGT

AAGCGGCGCGGGATTTCTGCCTCGCGGTGGTCATGCCCTTAATATGTAGC

ACTCAATTATCTGAGGCCTCATTCGTGAGGCCTCTTTACGTTTTTATGCGC

GAGTTCACTCCCGCATTCCCCGCGCTTAGCACAGCCACTCACCGTATTATT

CGCGATGCCCCGCTTCCCCCCTGCCGCGCTAGCGTCTCTCAGCTCAGTCAC

CGCATAGCCTTCGACCACGGCGATATCGACACGCGCGAAAAAATAAAAGC

CCGGCACATCCCGGGCTTTTATTTAAAATACTTATTCTGACGCGCCGCGTT

TATCTCTTTTCTCCGCCCGCCTTTCCTTCAGCGTCTTCTCTGCTTTTTTCTTG

GTGGCCTTATCCTTTCCTCTACTCATAAACACCTCGCTTA

>ET_sRNA_8_rev

TAGCAGATCGATAAATTCGTTGCGCTCGGCGTGCAGGAATGACCAACCGT

GCTGAATATGACGCAGCAGCGAGTGCGCCTTGTACTGGGTGATCACCCCT

ACGCGGCGCAGACCGGAGTTAATACAGTTGGAAAGGGTAAAGTCGATGA

TGCGAAAACGACTGCCAAAATAGACTGCGGGTTTGGCTCGGCCGTCGGTC

AGGCCATTAAGACGGGTTCCCCGTCCGCCGGCTAATACCAGCGCCACCGT

CCGTTGGGTTAATTGGCCGATAAGAGCAATATCTGTAACGCTATGAGTCA

TGGTCACTTCCTTTTTGACAATTCCATATGCCGCCCCGCCCACCGCCAGGC

AAGGGAAAGCGCGGATGGCGCCTGTCATCCGCAACAACAGTTTGGCGATC

CAGTATTAGCGTACTGCGGTGTAATAATAACGGACATCATTGATATAAAT

GAAATAAACTGAATGCTTATTTAATACCAAAATAATACGTTAACTGCTCAT

GTCAATCACACACTGATCGATAATGTAAACGCACTCTTTTTTAAATTCAAC

GAGAGATTGTGCTATTGCGCAGGATGCTGGAATAAACGGGTAGCGCAAAA

TATTACTTATATGGATAACATCTGCTAAATTAGCGTTAAATTTGAAAGTGG

ATATATATTGGTATTAAGCTGGGATAGGTTGATTTATAAGGGGGAATGTTT

TTTCTTAGCATAAAAAACGGAAGTTGCAAATAAATCACGGCCGTTTTTTAT

CGGCCGTGATTTATTGTTTTAGCCGCTAGCCCGCGGAGACTTTTTTACGGC

GCTGGTCCAAATCTTTTATCAATCGATTGATTTGCATATTATTAAACATAT

CCTCAAGCGTCATGTTTAGCTTGCGGCGCCAGTTCGGGTATTCATCGCAGG

TGCCGGGAACGTTGACCGGCGTCGCCATGTTCAGCCAATCCTCCGGCTGC

AGTCCGAGCAGGGCGCTGGCGCTGTCGGCGACGTAGCGTTGGATCCCGCG

GCTTAGAACGGGTGTCATCGCCATACGGTCCGCGTGCCGTCCGCTGCGCT

GCGGCAGGCAGCCGTAGTG

>ET_sRNA_9_rev

TAATGTATGGCATAATTAAACGTCACAACTCGTATCACCTACCGATGGGT

AAAGCGTGCACCACCGATACACCTCACCATCAATGATGTCAAGGCGATTT

CCCACGTGATTAGAATAATGTTATTTTTCACCCACCCCTCTGCACAAATTA

TTCCGTTTCCGCAAATCCGAATAAACCGCTGAGCGATAGTCGCCCTCCCAT

CCGCGTGAAAAATCACATTTTTTGAATAATTACACTACCAGAGCACGATA

TGCCGCGTATCACTGACCAACTACGCCCGGCAGGACCGGGCATTTAGCGC

GCTATCGATGTTTGCAACGATGTAAAGGCATGGATATTCAATTACTGATA

ACGCCCTCTTTAAGTATTCCCATTCAACCCGCAAGGCGAATTATATAAAGC

GTTCTATCTTTTCTATTCTGGCCGGTATGGCCATCATGTCACTGGCCGGAT

GCCCGCATCAACGCCCATTGCCACCCATCGAAACGGCTGTAGATAGCCAG

TATTAACATGACAAATCATGCAGTGACGTGTAATGCATCGCAGAAAGCCC

CCGTAGACTGGGCCATTGGAATTAAGGGTAATACACAAGCCAATGGCGCC

AAGGTGACTCAGAACCACCATCCTGATGGTTTTTTTACTTTTATCGTTAC

CAGGAATCAGCCTCAGGTGACTCAAGCGGCCATCCTTTAGGGTGGCCGCT

TCGCATCTATCGCCGCTTTTTAAGCGAGAGACATAAATTAACGATAAAGG

TAGTTAATCCAGGGTAAACAGATAAATAGATATGCCAACAGATTGACCAG

GCCAAATATGGCACCCAGCCGCCAAAAGTCTTTAGAGGGAAGATAGCCGC

TGCCGAAATAGATTGGGCTTGGCCCCGTTCCATAGGGCGTGATAATCCCC

ATGATCCCCAATGACAGAGAGATAGCCAGAGAAA

>ET_sRNA_10_rev

CCTTGATATGCCCCCTCATCGCGATAGCGGCTGAGCGAGAAGCGGCAGGC

GCCTATCCTGCCGGGATAACCGGGAAAAGAGAGCCACGTAACGCCCTGAA

TAAAATCCGCATCATCCCCCTGCTCGGCCCCATCGCGGGACACTCGGGGC
ACAATACTGCCGGAGGAAGAACGCATAAAAACCTCGTAATGGGTAAGAC
GTCATGGATATCGCCCCCCATAGTAGCAGCGAAGAGCACCCGCACTCACG
GTCGGTGAGCGCCTCCGATATCAAAAAATGAGCAAAACCGATCGATCCCC
GAAATATCAGCGCGCAGTACAACGCGGATGTCATGCTCGGCGGTGGTGCT
TCCCTCTGAACGATAGTAACCCAGGTTGTCTTCGACATATTGAACGATGAC
GCGCTGCGATCATCCGCGATGCGGCGTGGACGCTCACCCCGCCTTTGCCG
CCGTCAATACCTGCGACACCTTAGGCCGGCGCATCGGCGGAATATCCATC
AGCGATGCGCCGTTATCTCGGGGATCGATCGGTTTTGCTCATTTTTTGATA
TCGGAGGCGCTCACCGACCGTGAGTGCGGGTGCTCTTCGCTGCTACTATG
GGGGGCGATATCCATGACGTCTTACCCATTACGAGGTTTTTATGCGTTCTT
CCTCCGGCAGTATTGTGCCCCGAGTGTCCCGCGATGGGGCCGAGCAGGGG
GATGATGCGGATTTTATTCAGGGCGTTACGTGGCTCTCTTTTCCCGGTTAT
CCCGGCAGGATAGGCGCCTGCCGCTTCTCGCTCAGCCGCTATCGCGATGA
GGGGGCATATCAAGG
