# Supplementary figures and images for: Identification and validation of sRNAs in Edwardsiella tarda S08
Source: PLoS One. 2017 Mar 7;12(3):e0172783. doi: 10.1371/journal.pone.0172783 (PMC5340389; doi:10.1371/journal.pone.0172783)

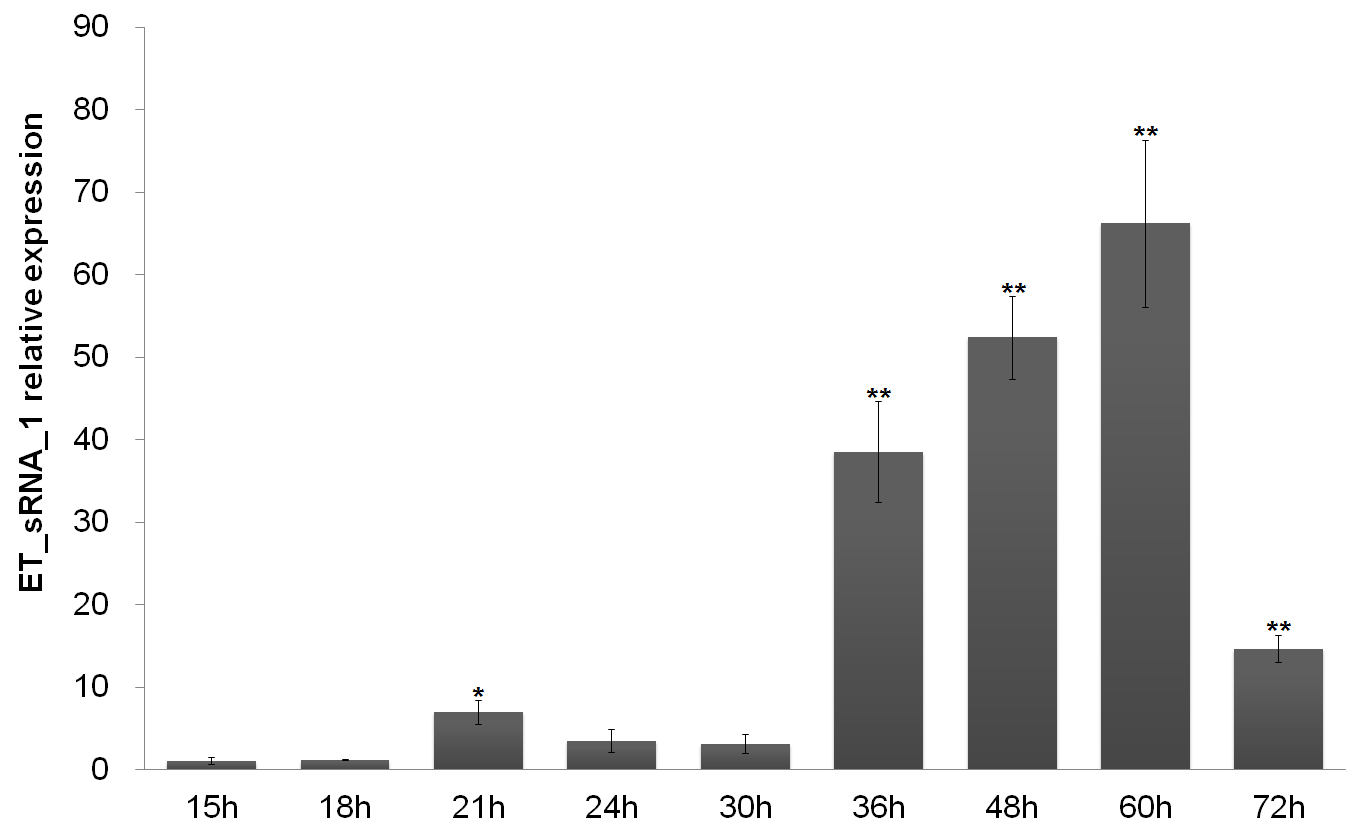

Supplement: S1 Fig — Statistical significance (*P≤0.05;**P≤0.01) was obtained using Anova test. (TIF) [file pone.0172783.s003.tif]

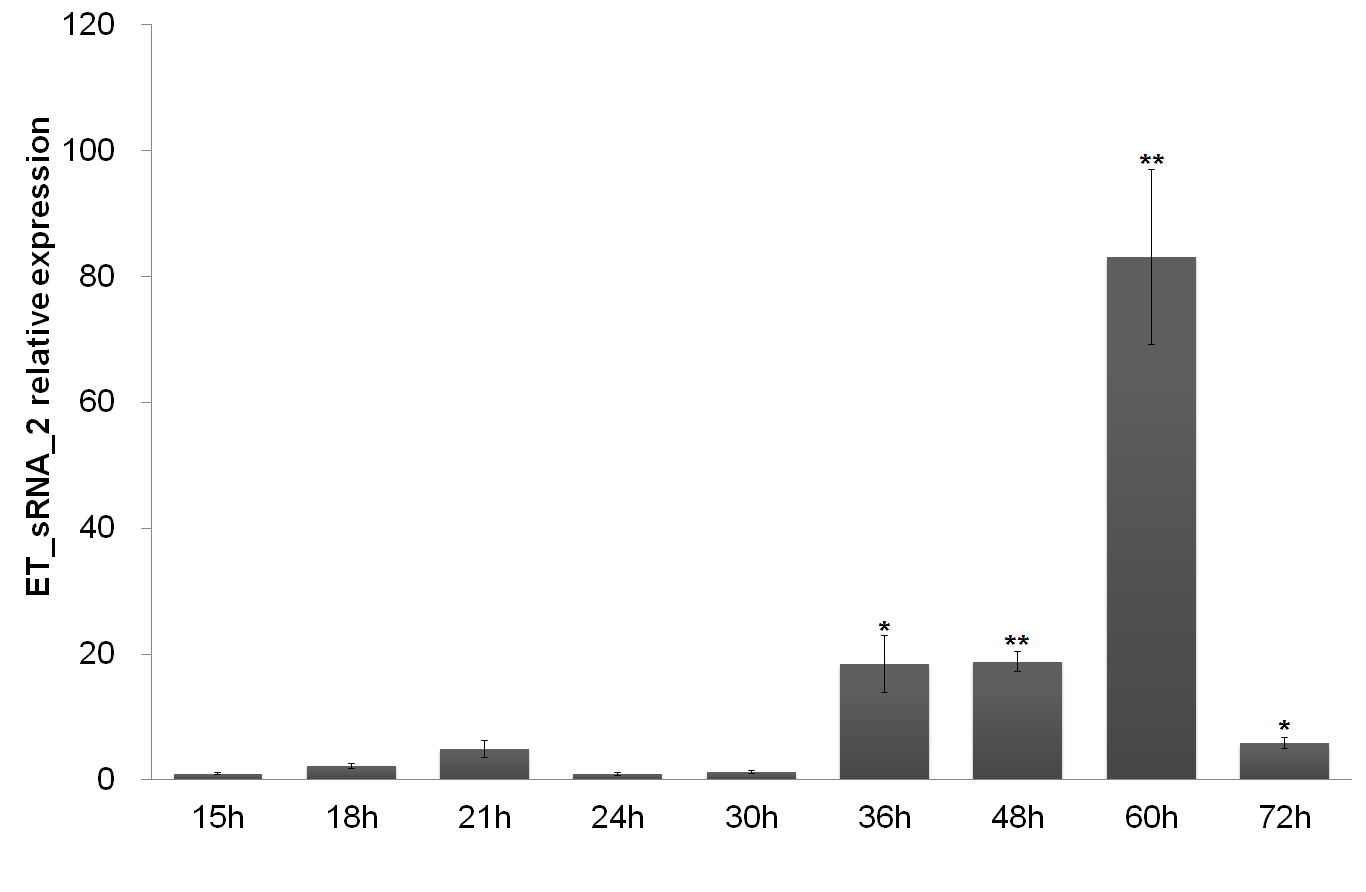

Supplement: S2 Fig — Statistical significance (*P≤0.05;**P≤0.01) was obtained using Anova test. (TIF) [file pone.0172783.s004.tif]

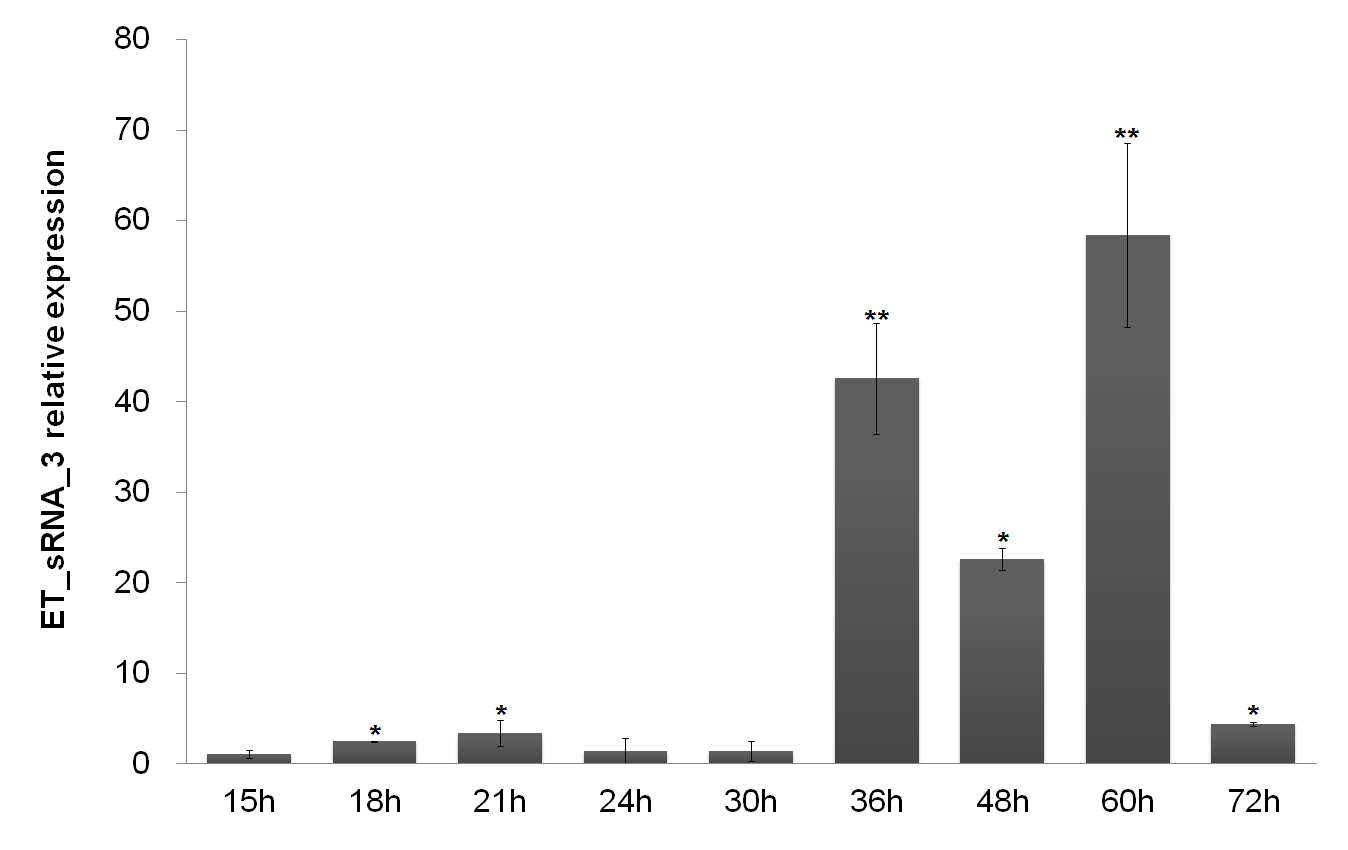

Supplement: S3 Fig — Statistical significance (*P≤0.05;**P≤0.01) was obtained using Anova test. (TIF) [file pone.0172783.s005.tif]

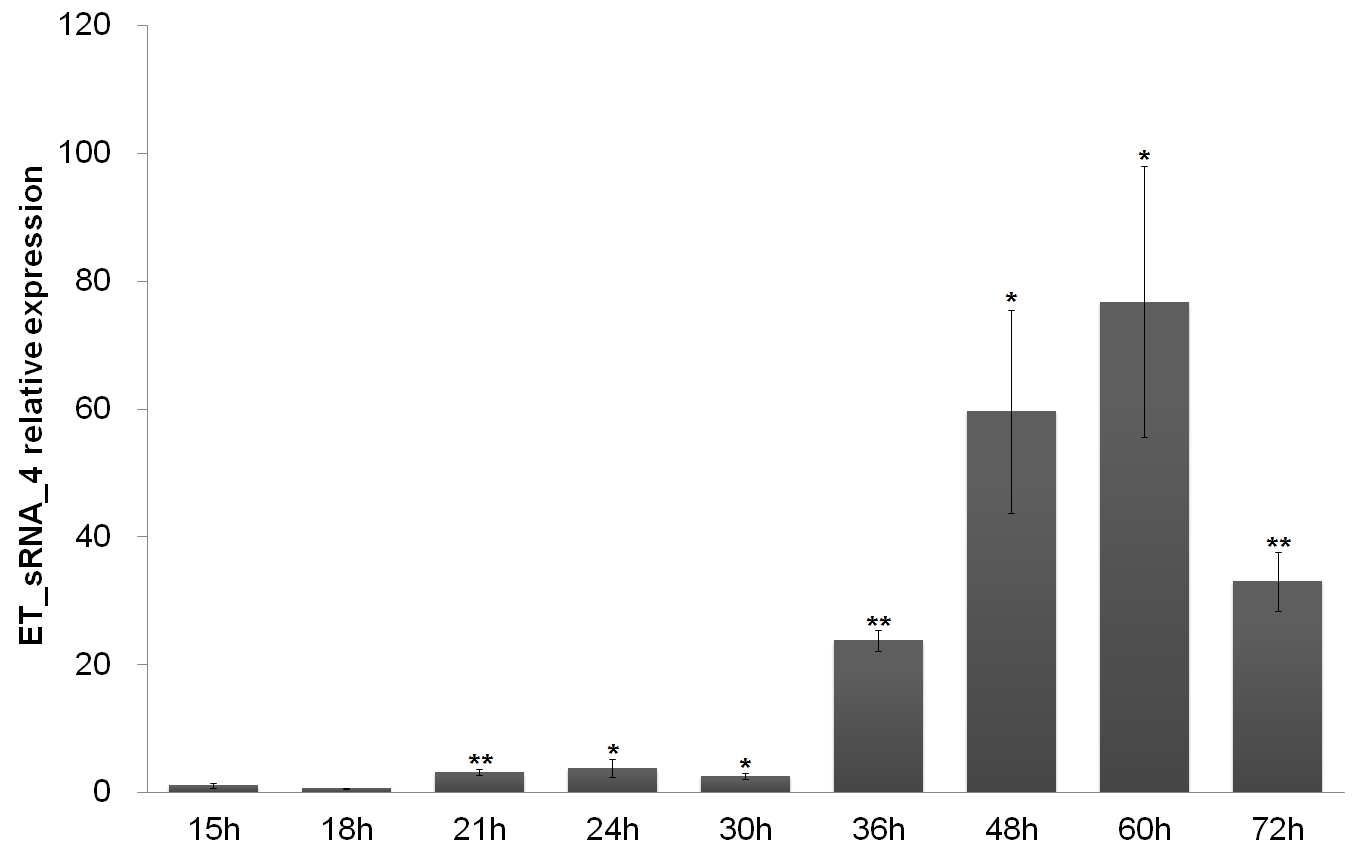

Supplement: S4 Fig — Statistical significance (*P≤0.05;**P≤0.01) was obtained using Anova test. (TIF) [file pone.0172783.s006.tif]

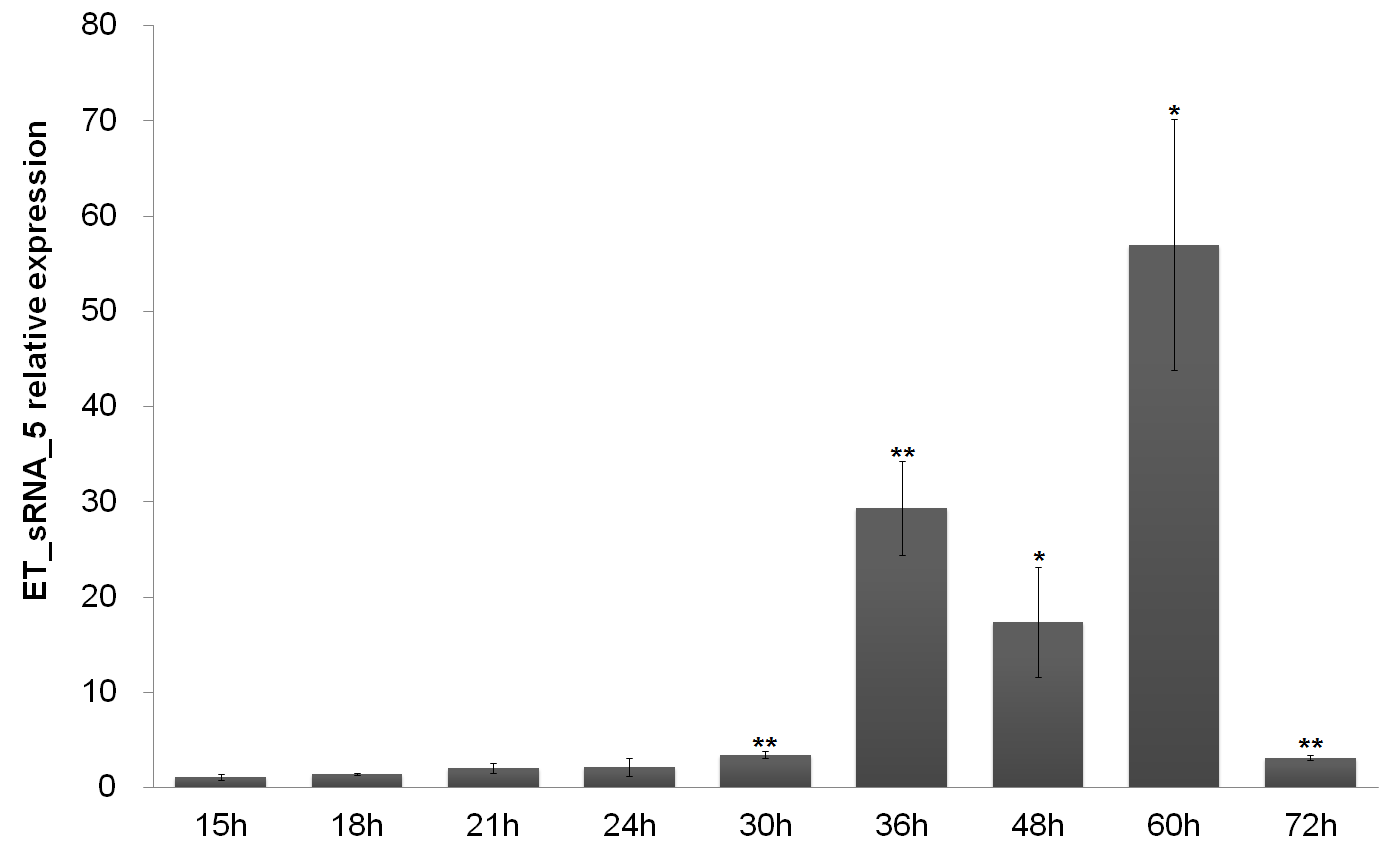

Supplement: S5 Fig — Statistical significance (*P≤0.05;**P≤0.01) was obtained using Anova test. (TIF) [file pone.0172783.s007.tif]

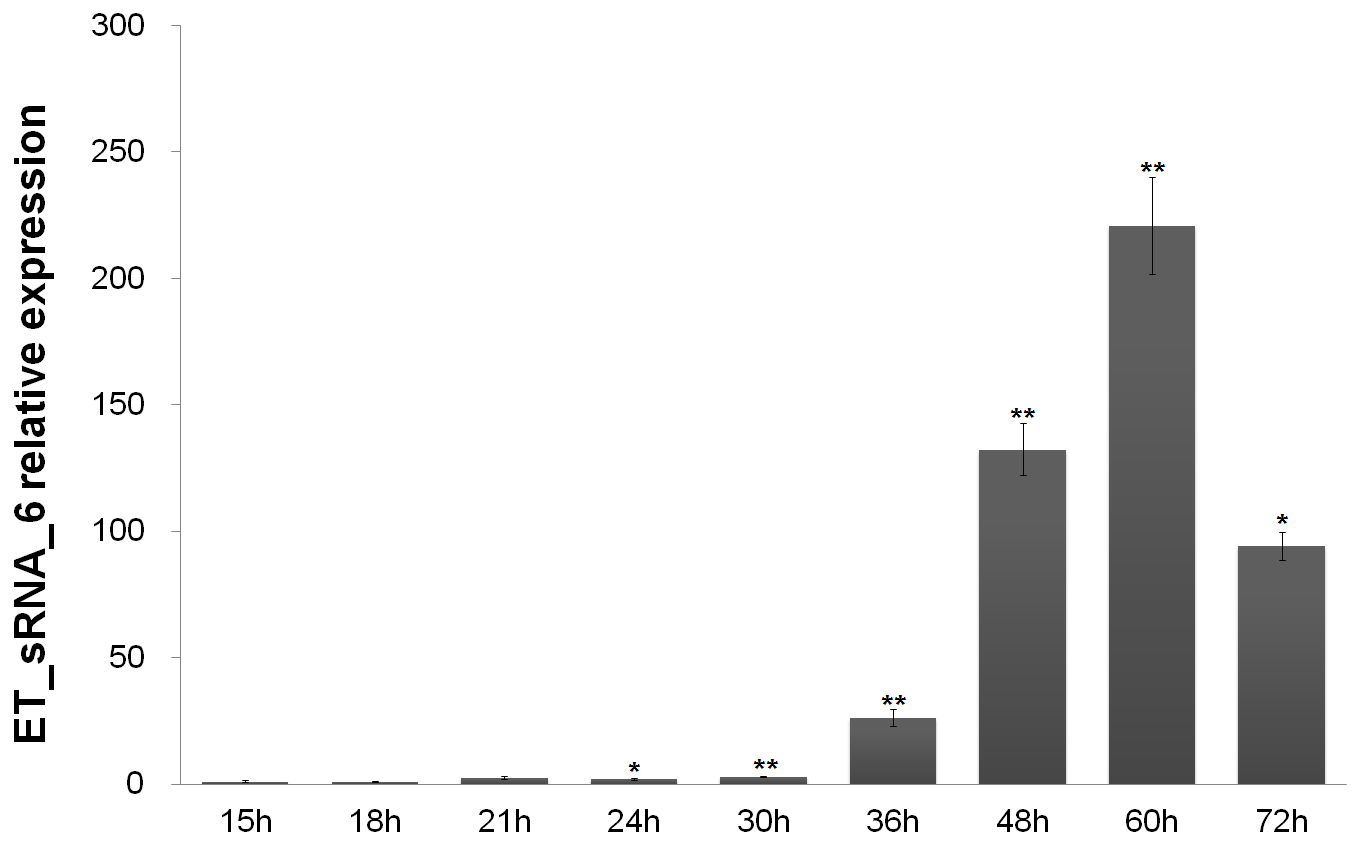

Supplement: S6 Fig — Statistical significance (*P≤0.05;**P≤0.01) was obtained using Anova test. (TIF) [file pone.0172783.s008.tif]

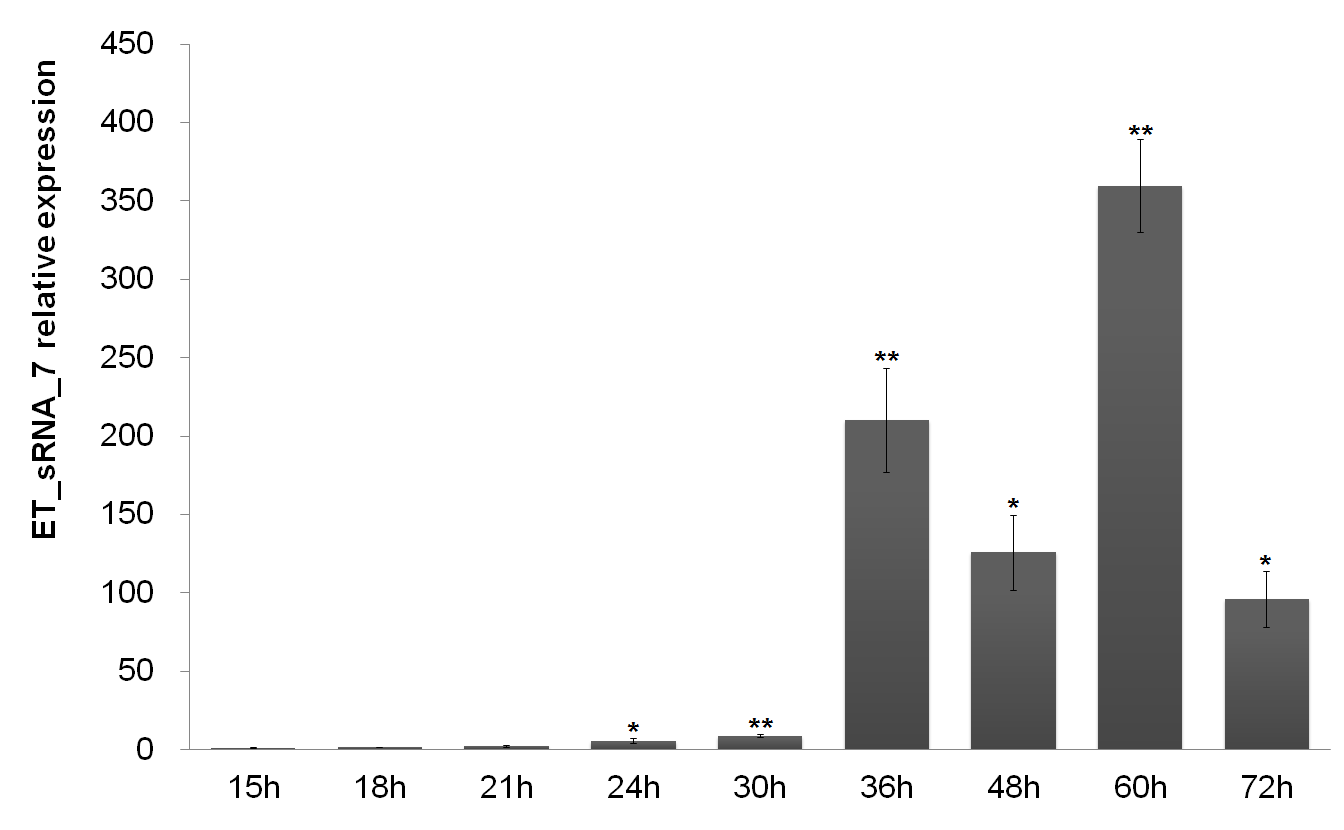

Supplement: S7 Fig — Statistical significance (*P≤0.05;**P≤0.01) was obtained using Anova test. (TIF) [file pone.0172783.s009.tif]

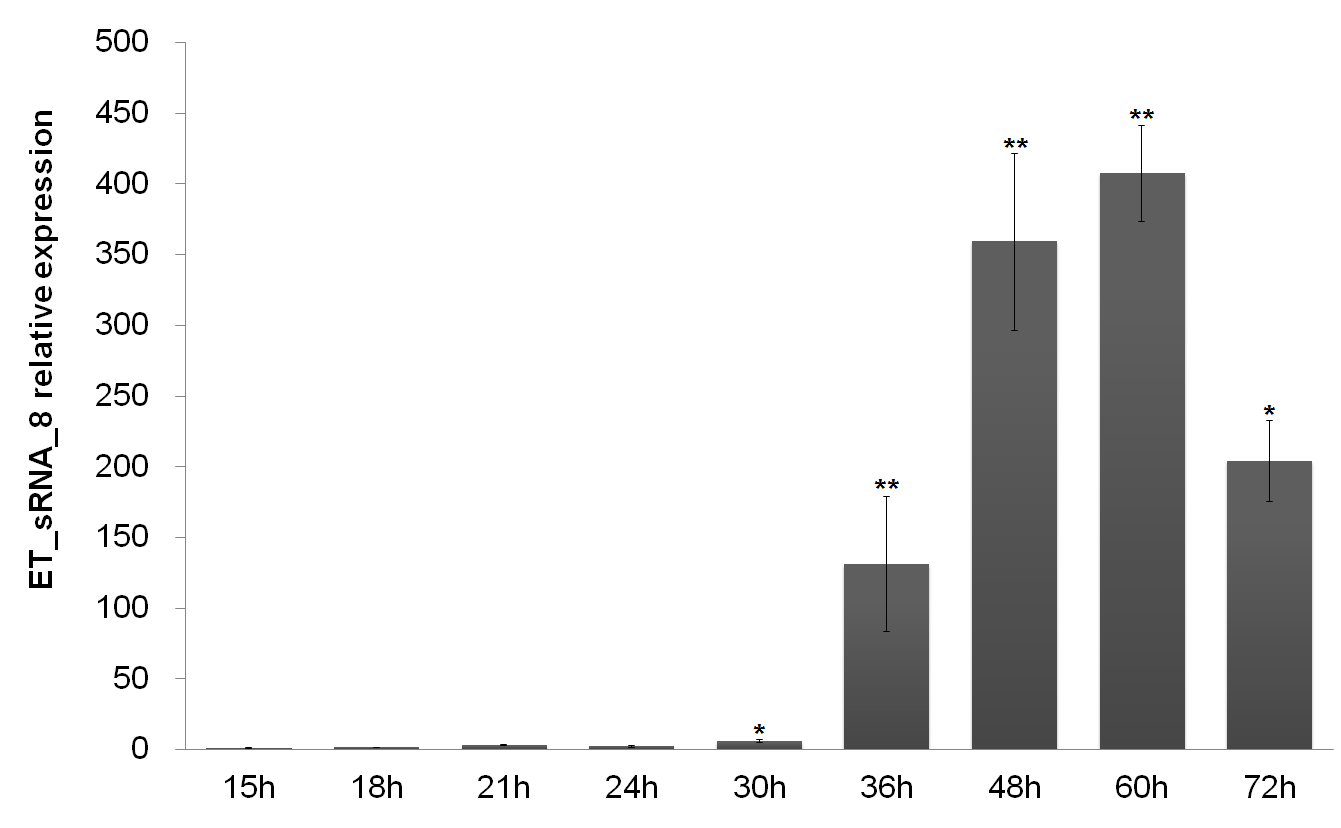

Supplement: S8 Fig — Statistical significance (*P≤0.05;**P≤0.01) was obtained using Anova test. (TIF) [file pone.0172783.s010.tif]

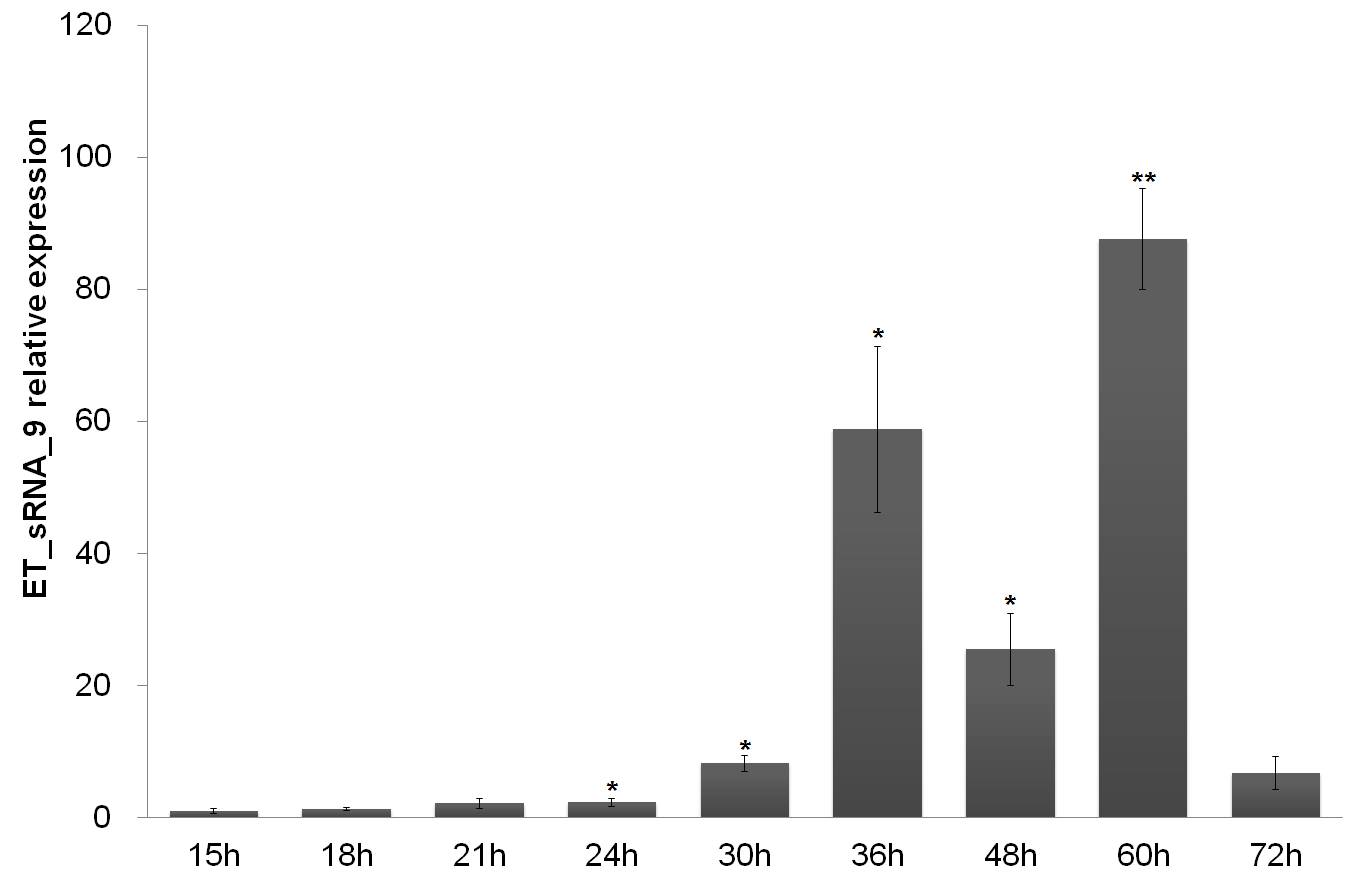

Supplement: S9 Fig — Statistical significance (*P≤0.05;**P≤0.01) was obtained using Anova test. (TIF) [file pone.0172783.s011.tif]
